# Imposed Work of Breathing for Flow Meters with In-Line versus Flow-Through Technique during Simulated Neonatal Breathing

**DOI:** 10.1371/journal.pone.0133432

**Published:** 2015-07-20

**Authors:** Snorri Donaldsson, Markus Falk, Baldvin Jonsson, Thomas Drevhammar

**Affiliations:** 1 Department of Women's and Children's Health, Karolinska Institutet, Stockholm, Sweden; 2 Department of Anaesthesiology, Östersund Hospital, Östersund, Sweden; University Children's Hospital Basel, SWITZERLAND

## Abstract

**Background:**

The ability to determine airflow during nasal CPAP (NCPAP) treatment without adding dead space or resistance would be useful when investigating the physiologic effects of different NCPAP systems on breathing. The aim of this study was to investigate the effect on pressure stability of different flow measuring devices at the in-line and flow-through position, using simulated neonatal breathing.

**Methods:**

Six different flow measure devices were evaluated by recording pressure changes and imposed work of breathing for breaths with 16 and 32 ml tidal volumes. The tests were performed initially with the devices in an in line position and with 5 and 10 L/min using flow through technique, without CPAP. The flow meters were then subsequently tested with an Infant Flow CPAP system at 3, 5 and 8 cm H2O pressure using flow through technique. The quality of the recorded signals was compared graphically.

**Results:**

The resistance of the measuring devices generated pressure swings and imposed work of breathing. With bias flow, the resistance also generated CPAP pressure. Three of the devices had low resistance and generated no changes in pressure stability or CPAP pressure. The two devices intended for neonatal use had the highest measured resistance.

**Conclusion:**

The importance of pressure stability and increased work of breathing during non-invasive respiratory support are insufficiently studied. Clinical trials using flow-through technique have not focused on pressure stability. Our results indicate that a flow-through technique might be a way forward in obtaining a sufficiently high signal quality without the added effects of rebreathing and increased work of breathing. The results should stimulate further research and the development of equipment for dynamic flow measurements in neonates.

## Introduction

Measurement of airway flow of a spontaneous breathing infant allows determination of respiratory parameters during respiratory support and forms the basis for lung function testing. A technique suitable for neonatal use requires minimal resistance and dead space to avoid significant effects on breathing [1, 2]. The quality of the flow recordings also needs to be high.

### Resistance

The resistance of a flow meter adds to the imposed work of breathing (WOB). This may change the infants breathing pattern and affect measurements. This is of particular concern in studies of imposed work of breathing and also for variables that are affected by increased resistance.

The most common neonatal respiratory support is nasal continuous positive airway pressure (NCPAP). The resistance to breathing and imposed WOB of these systems show large variations [3, 4]. Further, imposed WOB has been suggested as a contributing factor that may affect clinical outcome. Measuring flows without adding imposed WOB would therefore be a valuable tool in clinical studies using different CPAP equipment. Combining low imposed WOB NCPAP systems and high imposed WOB measuring techniques will generate results that are difficult to interpret.

Imposed WOB can be determined in mechanical lung models by analysing the pressure changes during simulated breathing. Systems with higher resistance will display larger pressure swings and are less pressure stable. The imposed WOB represents the area of a pressure volume loop. To allow comparisons the volume has to be constant and therefore these experiments cannot easily be performed in animals or humans.

### Dead space and the positioning of the measurement device

**Fig 1:**
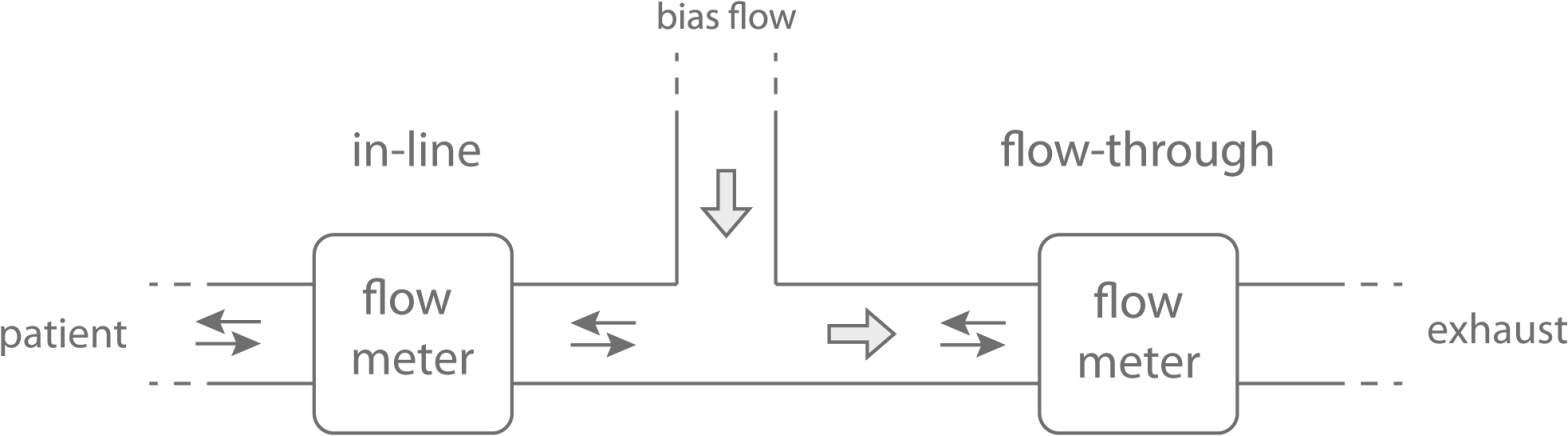
In-line and flow-through position. With the in-line position the flow meter simply records patient breathing but dead space will be increased. In the flow-through position the flow meter records the breathing offset by the added bias flow. If the bias-flow is not constant a second flow meter is needed to measure the bias-flow offset. If the bias flow is sufficient the volume of the flow meter will not add to dead space.

Infants are sensitive to the addition of extra dead space and the resulting CO2 increase will affect breathing. There are two positions where flows can be measured (Fig 1). The *in-line position* (I) measures breathing directly, with the flow meter attached to the endotracheal tube or patient interface. The volume of the measuring device will add to dead space. The *in-line* position has been the standard technique used in studies reporting results from neonatal resuscitation and pulmonary function testing [1, 5, 6]. The *flow-through position* (II) measures breathing after a bias-flow has been added between the measuring device and the patient [7]. The volume of the measure device does not add to dead space if the bias flow is high enough to prevent re-breathing. The main disadvantage with this technique is that it is technically more difficult because the added bias flow has to be subtracted. The subtraction makes the flow-trough position sensitive to non-constant bias flow and drift of the flow meter. Modern adult and pediatric ventilators use flow-through techniques when they measure flow on the expiratory limb of a breathing circuit. The flow-through technique using a constant bias-flow was first described by Rigatto and Brady [8] and has been used in spontaneously breathing infants in several studies.

The importance of dead space and low resistance to flow is thoroughly discussed by the ERS/ATS task force on Standards for Infant Respiratory Function Testing [1].

### Quality of measurements and flow range

The quality of flow measurements has to be sufficiently high to accurately record flows and then allow integration to enable the calculation of volumes. The flow range that a device is designed for has a balance between flow resistance and the ability to obtain high quality measurements for the intended measurement range. It is difficult to achieve accuracy at low flows but can be compensated for by reducing the measuring cross sectional area. At higher flows a reduced area can be problematic due to the risk of adding increased resistance. The balance between adding resistance (increasing work of breathing) and improving accuracy will be more delicate as tidal volumes and flows are reduced. An extreme example of this is a spontaneously breathing extremely premature infant that is approaching respiratory failure.

The sampling rate has to be sufficiently high to measure fast breathing and allow for signal processing.

There are several techniques and devices available for measuring flow. These include hot-wire, chip-based mass flow meters, pneumotachographs and ultrasonic flight of time techniques. Hot wire techniques are based on the cooling effect that flow has on heated wires. Chip-based sensors also use cooling but on a surface instead of wires. Pneumotachographs measure flow by the pressure difference generated over a resistor. Ultrasonic flight of time is based on changes in time that it takes a signal to travel a distance with or against flow. The devices have different properties and vary in external size, dead space, resistance, requirements for ongoing recalibration and current clinical use.

Several of these factors will be important for clinical use even if this study focused on flow meter resistance and positioning of the devices. There are no studies in the literature comparing imposed work of breathing for different flow meters or comparisons of the effect of in-line and flow-through placement.

The aim of this study is to investigate pressure stability for different flow measuring devices with the in-line and flow-through position, using simulated neonatal breathing.

## Materials and Method

**Fig 2:**
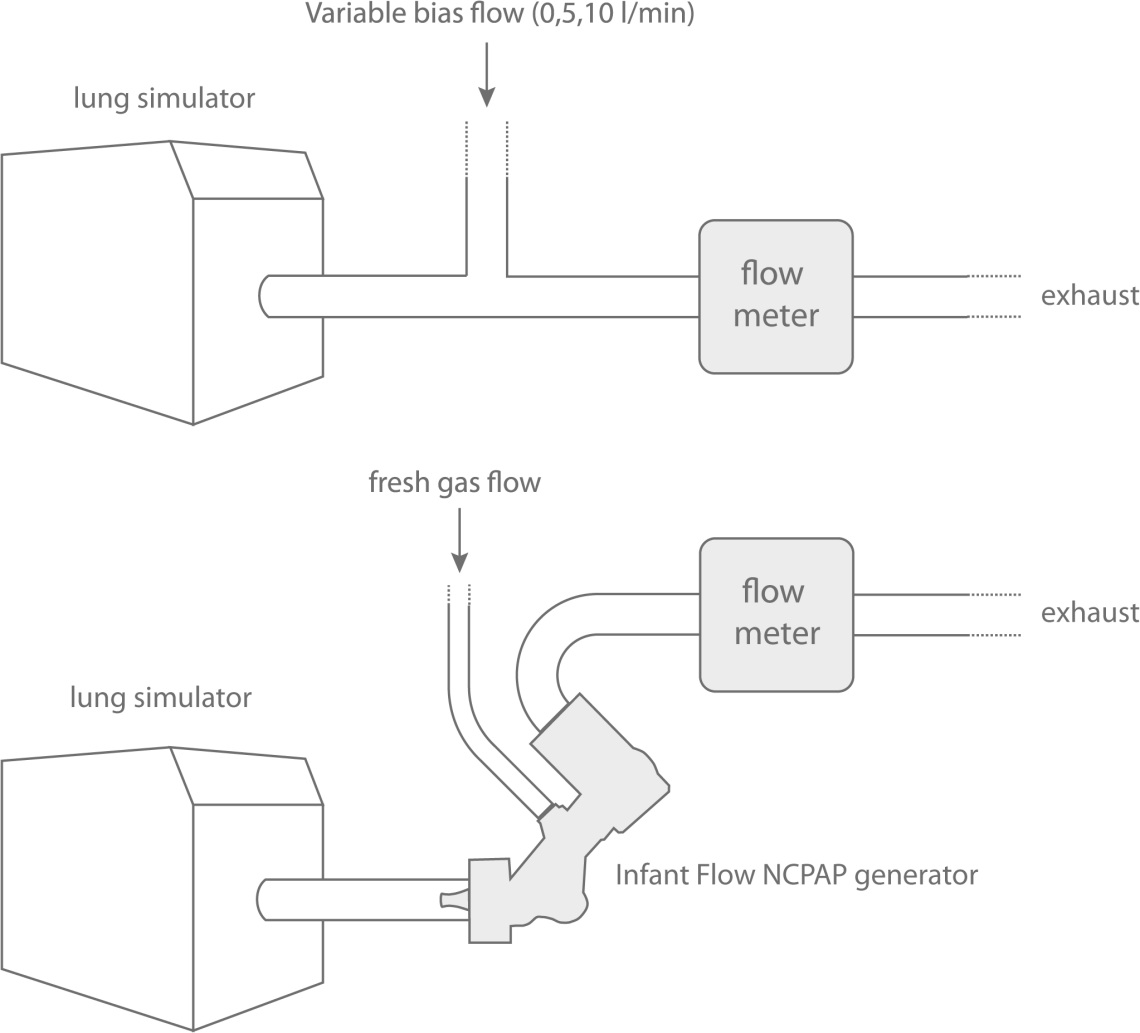
Experimental setup. The *bias flow experiments* (top) were performed to measure pressure stability and imposed WOB with in-line and flow-through position. A bias flow of 0 L/min is equivalent to in-line positioning. The *variable flow CPAP generator* experiments (bottom) were performed with an Infant Flow variable flow NCPAP generator. To simplify, the pressure recording tube on the Infant Flow was not included in the illustration.

The flow meters (Table 1) were tested in two experiments; first in a bias flow experiment (no CPAP) and then in combination with a NCPAP system (Fig 2).

**Table 1:** Characteristics of the tested flow meters.

Flow meter	Principle	DAQ	Dead Space	Resistance
Fleisch 0 (Metabo, Epalinges, Switzerland)	Pneumotachograph 10 mm ID	Differential pressure transducer and AD conversion	4 ml	0.04 cm H2O/(L/min)
Vitalograph Fleisch (Vitalograph Ltd. Buckingham, UK)	Pneumotachograph 50 mm ID	Differential pressure transducer and AD conversion	>50 ml^a^	<0.017 cm H2O/(L/min)
FLORIAN (Acutronic Medical Systems AG, Hirtzel, Switzerland)	Hot-wire sensor (53000–0101)	Analog output and external AD conversion	1 ml	Not stated^b^
EXHALYZER S (ECO MEDICS AG, Dürnten, Switzerland)	Ultrasonic with size S dead space reducer (DSR)	Dedicated software	1.9 ml	Not stated^b^
SFM3200 prototype (SENSIRION AG, Staefa, Switzerland	Chip	Dedicated software	<21 ml	0.014 cm H2O/(L/min)
SpiroQuant A (EnviteC-Wismar GmbH, Wismar, Germany)	Hot-wire	Dedicated software	15 ml^a^	Not stated^b^

Resistance according to manufacture recalculated to H2O/(L/min).

^a^ Estimated dead space volume.

^b^ The resistance is not stated by the manufacturer.

The *bias flow experiments* were performed with a lung simulator (ASL 5000, IngMar Medical, Pittsburg, PA). The flow meters were connected to the simulator with a T-piece and an adjustable bias flow added between the lung simulator and the measuring device. Three levels of bias flow were tested: 0, 5 and 10 L/min. The flow was constant and measured by VT PLUS HF (Fluke Biomedical, Everett, WA). In-line position refers to, and is equivalent to, a 0 L/min bias-flow. Flow-through technique refers to the experiments with a bias-flow. The systems were tested with two sinusoidal flow patterns with a maximum flow of 3 and 6 L/min (16 ml and 32 ml tidal volume) and a respiratory rate of 60 /min. The flow pattern was symmetrical with identical inspiration and expiration. The results in the bias flow experiments reflect the resistance of the flow meter.

The *variable flow CPAP generator* experiments were performed with an Infant Flow system (VIASYS, Palm Springs, CA) and medium prongs. Recordings were performed at 3, 5 and 8 cm H2O CPAP. The system was attached to the lung simulator with a straight 22 mm connector. The flexible exhaust tubing was replaced with 20 cm (10 mm ID) silicone tubing and the flow meter attached at the distal end. The flow generating CPAP left the system through the exhaust tubing and used the flow-through technique (CPAP driving flow was equal to bias flow in the first experiment). No in-line measurements were performed with the NCPAP system. The systems were tested with a symmetrical sinusoidal flow pattern with a maximum flow of 6 L/min (32 ml tidal volume, respiratory rate 60 /min). The results in the variable flow CPAP generator experiments reflect the resistance of both the Infant Flow system and the flow meter. To allow comparison, data from the Infant Flow system without attaching a flow meter was included.

The different flow meters were used with their dedicated hardware and software. For devices with an analog output the signal was converted using an analog to digital converter (LabJack U6, StreamLD version 1.12, sample rate of 250 Hz and streamed to a file). For those systems that needed calibration, this was performed with a Fluke, VT PLUS HF. All simulations were performed with dry, unheated air.

The measured variables were delivered CPAP pressure, pressure swings and imposed work of breathing, for each breath. Mean values were calculated for 20 consecutive breaths (number 10–29). The iWOB were presented with a 95% confidence interval in graphs. Complete data including variance was uploaded as supplemental information. Flow recordings from the devices were compared graphically and presented as examples of data from one breath without any filtering or signal processing.

The delivered pressure and imposed work of breathing was recorded and calculated in the mechanical lung model (ASL 5000 modified software 3.1). The lung model consists of a servo-controlled piston and the pressure is measured inside the lung model. The volume is given by the position of the piston and corrected for gas compression. The iWOB was calculated by integration of the pressure-volume loop for each breath and presented as mJ/breath. The integration was done for inspiration and expiration separately and then added to give the total iWOB for one breath. For each breath the CPAP, highest and lowest pressure in the lung model was recorded.

Data was compiled with Excel 2010 (Microsoft Inc., Redmond, WA) and processed in SPSS (ver. 21, IBM).) Original data was deposited for open access (DOI provided at the end of references). Statistical comparisons were performed with one-way ANOVA with Bonferroni correction. P <0.05 was considered statistically significant.

## Results

The resistance of the flow meters revealed effects on pressure stability in both the bias flow (in-line and flow-through technique) and the NCPAP experiments (Infant Flow with flow-through technique). The effects were larger with flow meters with higher resistance.

### In-line and flow-through technique

The differences between SpiroQuant A, SFM3200 and the Vitalograph Fleisch were very small. For example the differences in pressure swings or generated CPAP was below 0.05 cm H2O (not statistically significant in several comparisons).

**Fig 3:**
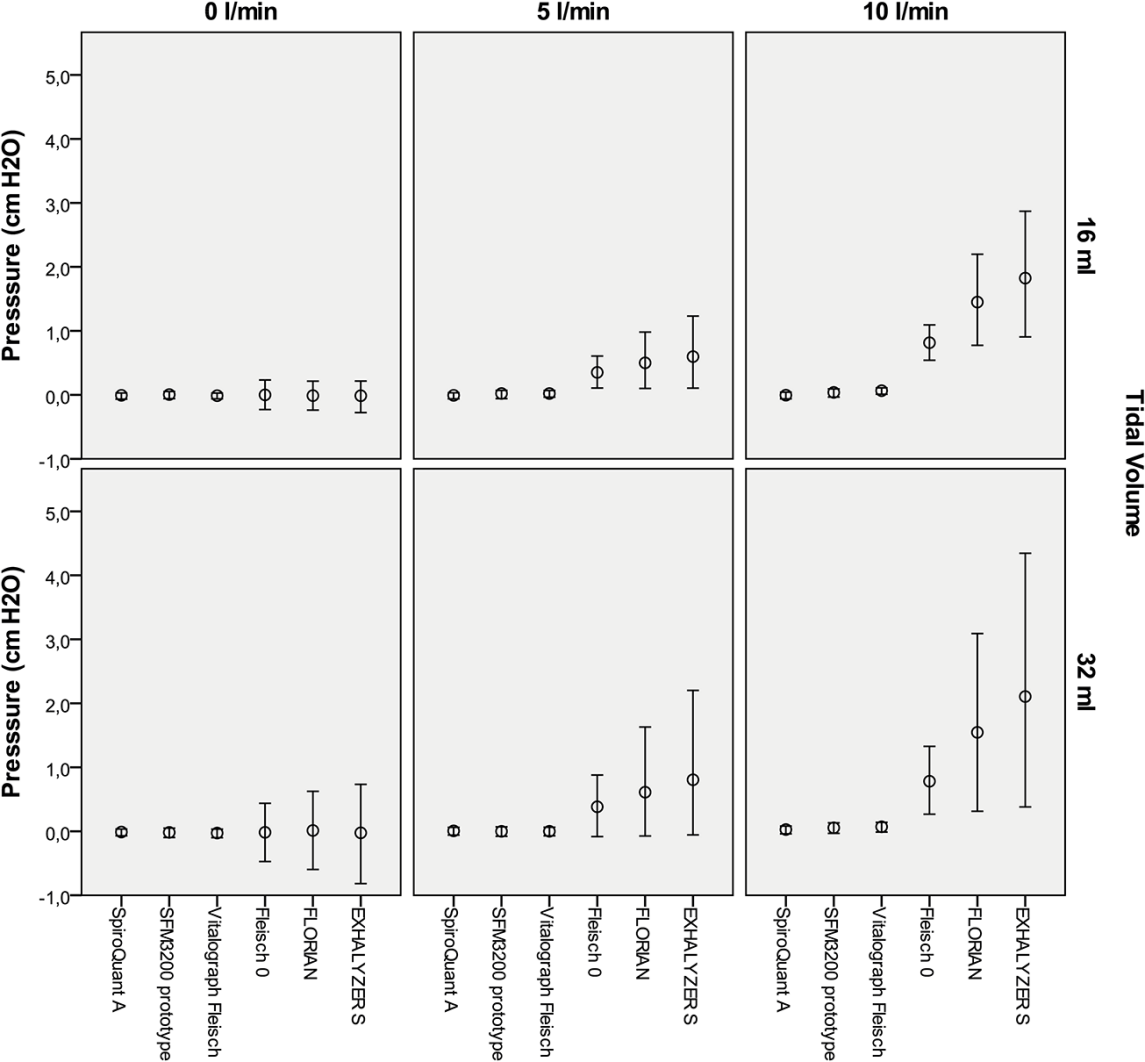
Pressure swings and generated CPAP for three levels of bias flow. A bias flow of 0 L/min is equivalent to in-line placement of flow meter. The circle indicates mean pressure (CPAP) and the bar the mean highest and lowest pressure (the amplitude represent pressure swing) for 20 consecutive breaths. Complete data including variance is available as supplemental information (S1 Table).

**Fig 4:**
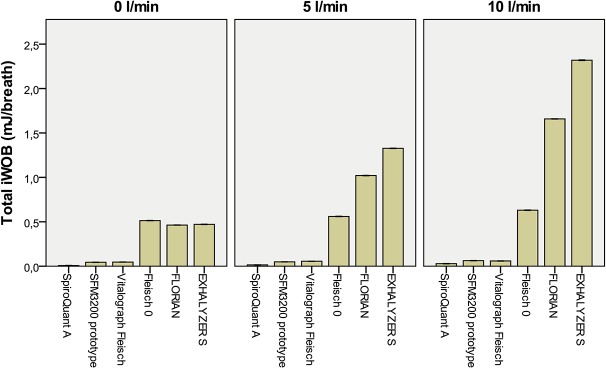
Imposed WOB (Total iWOB) at three levels of bias flow at simulated breathing with tidal volume 16 ml. A bias flow of 0 L/min is equivalent to in-line placement of flow meter. Bar indicate mean imposed WOB with 95%CI for 20 consecutive breaths.

**Fig 5:**
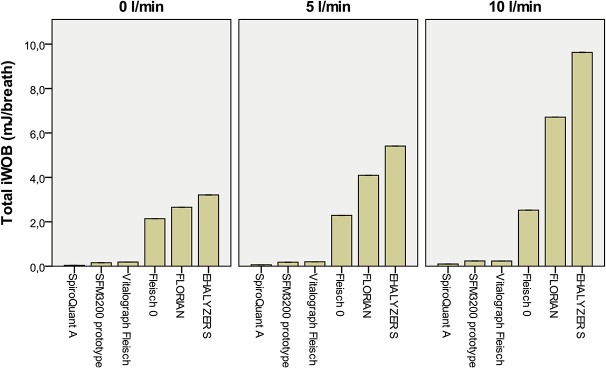
Imposed WOB (Total iWOB) at three levels of bias flow at simulated breathing with tidal volume 32 ml. A bias flow of 0 L/min is equivalent to in-line placement of flow meter. Bar indicate mean imposed WOB with 95%CI for 20 consecutive breaths.

The flow meters with higher resistance (Fleisch 0, Florian and NDD) showed larger pressure swings and increased imposed WOB (Figs 3–5). In simulations with bias flow they also generated CPAP. The generated CPAP and the increase in imposed WOB were statistically significant in all comparisons except for simulations with 0 L/min bias flow and low tidal volumes (16 ml) (not statistically significant in several comparisons).

Complete data and statistical comparisons are available as supplementary information (S1 Table).

### Infant Flow with flow-through technique

**Fig 6:**
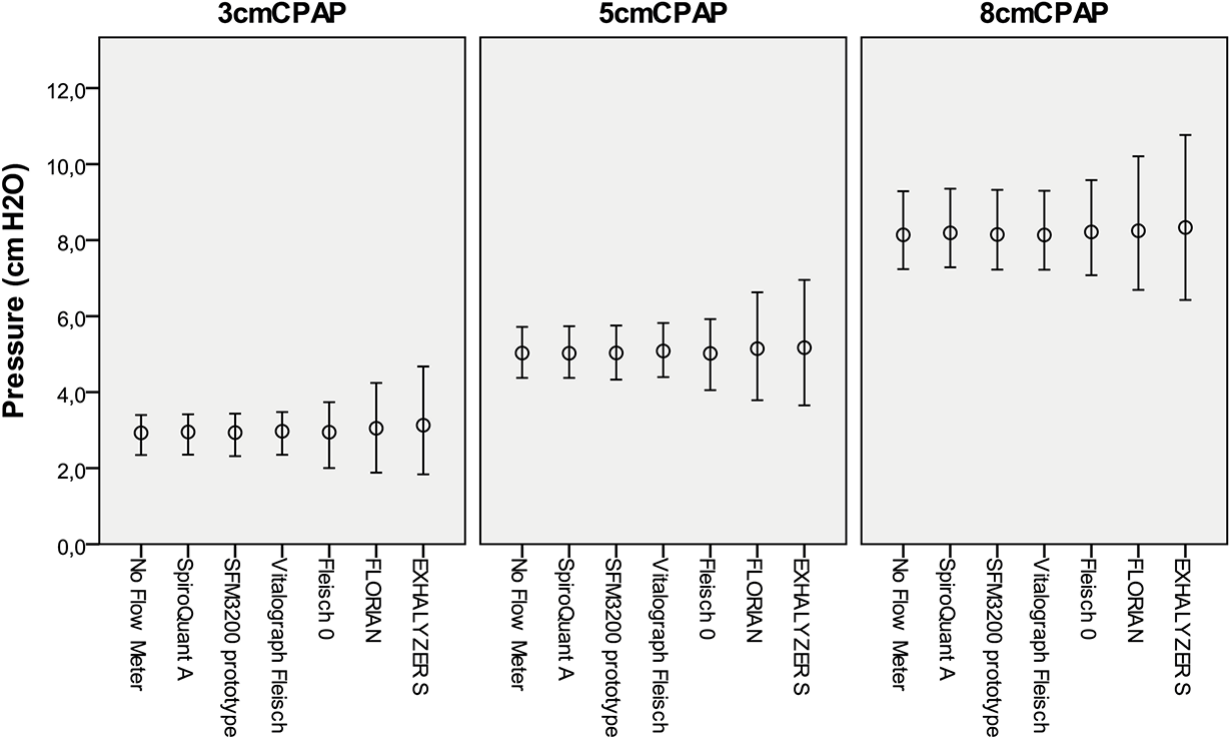
Pressure swings for Infant Flow with flow meters. Simulations using flow-through measurement at three levels of CPAP with 32 ml tidal volume breathing. The circle indicates mean pressure (CPAP) and the bar the mean highest and lowest pressure (the amplitude represent pressure swing) for 20 consecutive breaths. Complete data including variance is available as supplemental information (S2 Table).

**Fig 7:**
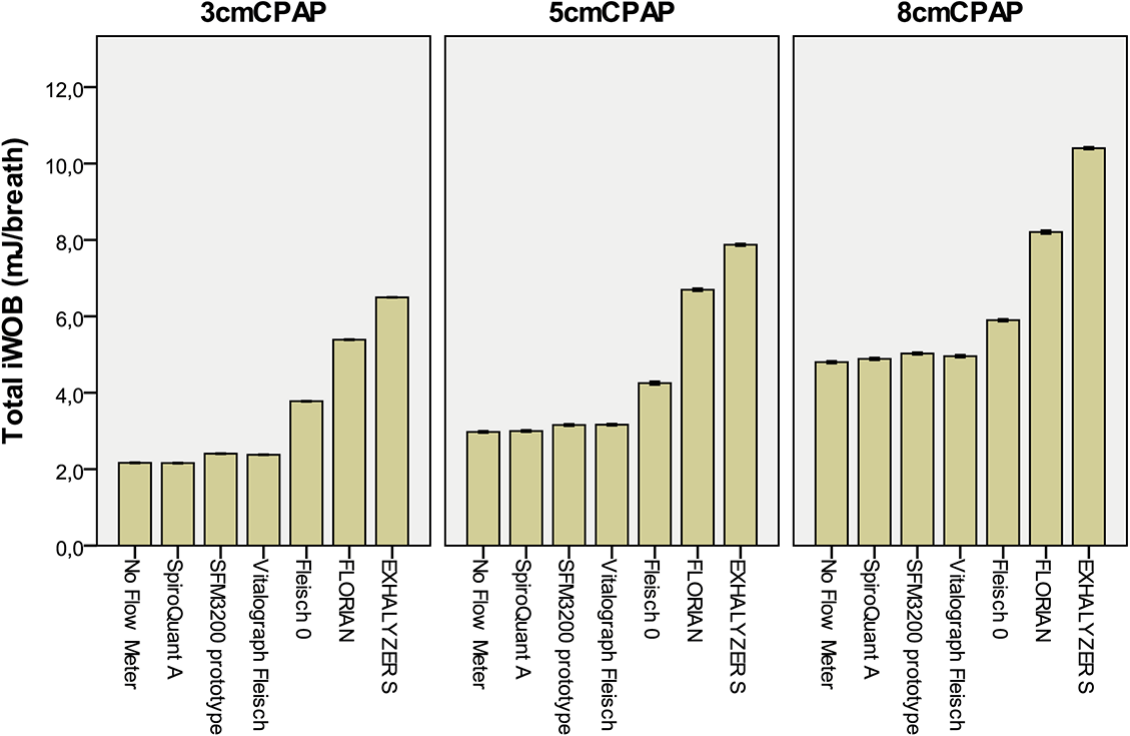
Imposed WOB for Infant Flow with flow meters. Simulations using flow-through measurement at three levels of CPAP for simulated breathing with 32 ml tidal volume. Bars show mean imposed WOB (Total iWOB) for twenty consecutive breaths with 95% CI.

The differences in pressure stability between SpiroQuant A, SFM3200 or the Vitalograph Fleisch pneumotachograph, in combination with Infant Flow, were very small (Figs 6 and 7). As an example, the differences in pressure swings were below 0.1 cm H2O (not statistically significant in several comparisons). Infant flow used with Fleisch 0, Florian or NDD increased imposed WOB and pressure swings (p <0.05 in all comparisons). Complete data and statistical comparisons are available as supplementary information (S2 Table).

### Quality of flow recordings

**Fig 8:**
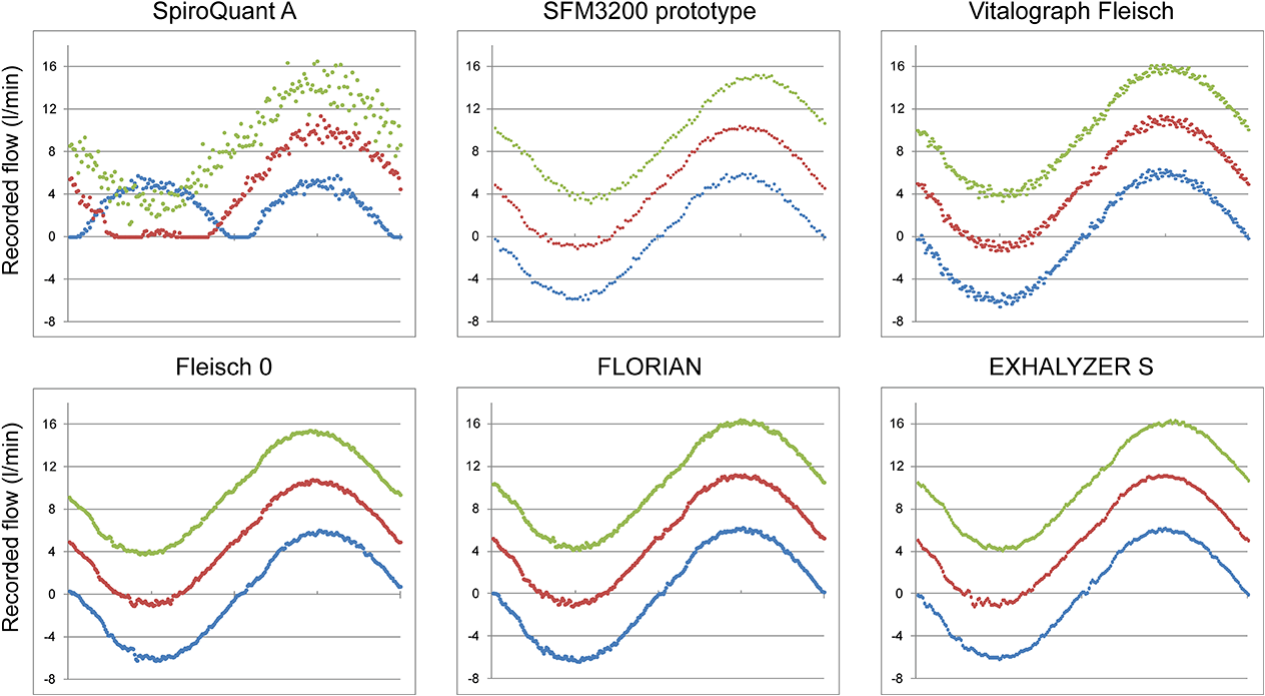
Graphic quality of signal at three levels of bias flow. Recordings from simulations at three levels of bias flow with simulated sinusoidal breathing (32 ml tidal volume, 60 breaths/minute). The colors indicate bias flow level; blue 0.0 L/min, red 5.0 L/min and green 10.0 L/min. The graphs represent one breath (1.0 s).

**Fig 9:**
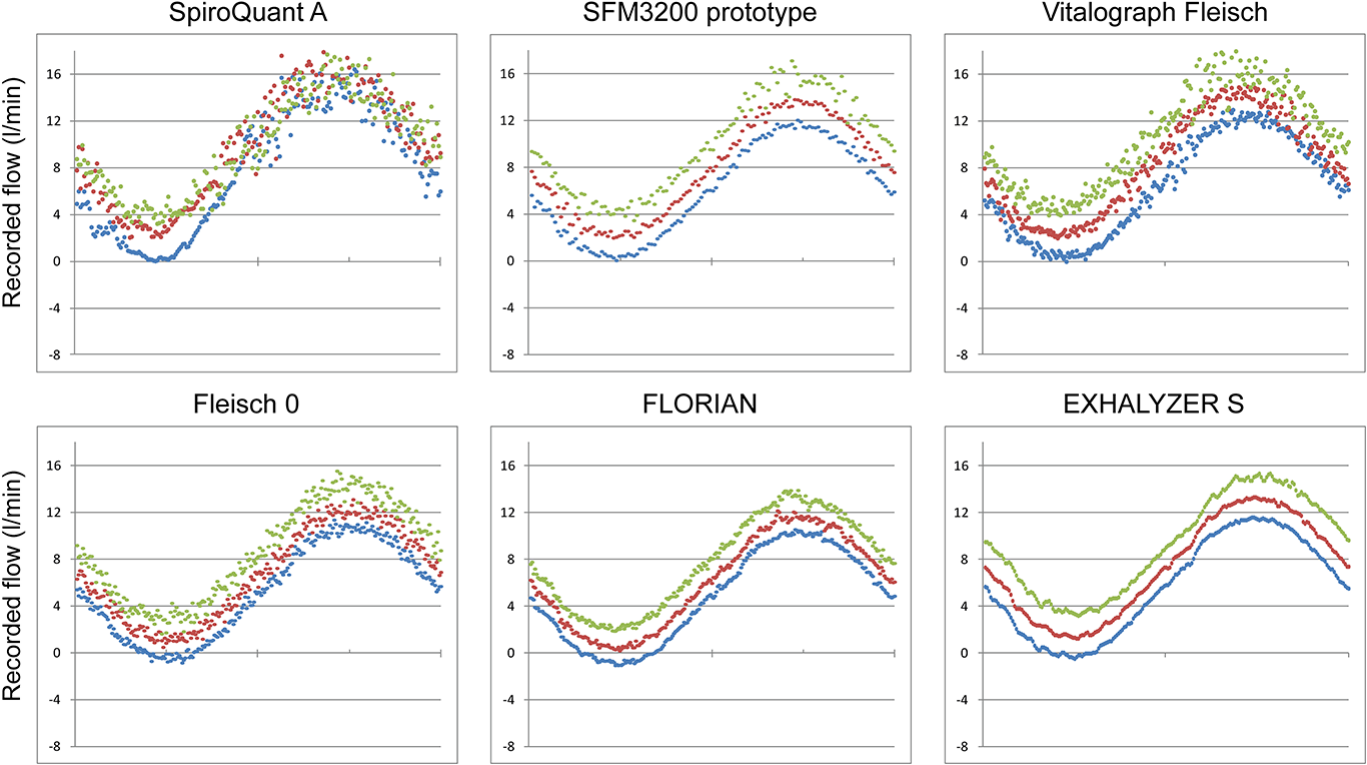
Graphic quality of signal at three levels of CPAP. Recordings from simulations with flow meters connected to the expiratory limb of an Infant Flow device at three levels of CPAP with simulated sinusoidal breathing (32 ml tidal volume, 60 breaths/minute). The colors indicate CPAP level; blue 3.0 cm H2O, red 5.0 cm H2O and green 8.0 cm H2O. The graphs represent one breath (1.0 s).

A graphical comparison between all flow recordings is presented in Figs 8 and 9. The SpiroQuant A had artifacts at flows close to zero. The other techniques showed similar graphic quality but with different levels of noise. All recordings from *Infant Flow with flow-through technique* showed increased levels of noise (Fig 9).

## Discussion

### Dead space and flow resistance

The use of in-line flow measurement will increase re-breathing because of the increase in dead space due to the volume of the flow meter. The low resistance devices tested in this study had a higher dead space and should therefore not be used in the in-line position. This would be of particular concern when measuring flow recordings with spontaneous breathing of longer durations. Systems dedicated for neonatal use, such as Florian, had a smaller dead space but higher flow resistance. The reported dead space volumes in Table 1 were static and the dynamic dead space may be smaller. This means that a clinical effect may be less than anticipated even if rebreathing will be a concern with larger dead space. Large differences between static and dynamic dead space during exhalation have been described during capnography in infants [9].

For in-line technique simulations (no bias flow and no NCPAP system) the resistance of Florian and EXHALYSER S generated an increase in pressure swings and imposed WOB compared to other devices but the absolute differences were small. For example the imposed WOB was 2.7 mJ/breath for Florian and 3.2 mJ/breath for EXHALYSER S (32 ml TV). This is at the same level as Infant Flow NCPAP at 5 cm H2O (3.0 mJ/breath, without flow meter). In the same simulations SpiroQuant A, SFM3200 and the Vitalograph Fleisch pneumotach had lower resistances and showed very low imposed WOB (<0.2 mJ/breath).

When measuring flows with flow-through technique, re-breathing and dead space is not relevant. This is because the flow meter is positioned on the expiratory side and the bias flow washes out the dead space [8].

The resistance of the flow meter will have two different effects when used with the flow-through technique 1) It will increase imposed WOB and 2) generate CPAP from obstructing the outflow. This was seen in both the experiments with bias flow and in the Infant Flow experiments. The effect was more pronounced at higher bias flows and for flow meters with higher resistance.

In simulations with Infant Flow at 5 cm CPAP the imposed WOB more than doubled when flow was measured with Florian or EXHALYSER S. The flow meters with low resistance showed no effect on imposed WOB or CPAP generation (Fig 7).

In summary the flow-through placement is insensitive to dead space of the measuring devices. The importance and effect of dead space will depend on the duration of measurement as well as system design and leakage. Both in-line and flow-through techniques were sensitive to flow meter resistance. Higher resistance increased imposed WOB and, in simulations with bias flow, generated a small level of CPAP pressure.

### Graphical comparison of flow recordings

The levels of signal processing were different between the flow meters. Systems like FLORIAN or SpiroQuant A included more sophisticated hardware and software compared to the pneumotachographs that relied on raw measurements of differential pressures. This makes detailed comparisons difficult and the graphical comparison has to be seen as an indicator of quality rather than a detailed test of accuracy or performance. The sampling frequency may reflect hardware settings rather than true limitations. The use of graphic quality can be used as a way to screen flow measurement but it can only be regarded as the first step towards clinical use. Clinical use demands further optimization and validation of signal quality as well as safety and usability.

The recordings with bias flow showed some clear disadvantages. The bias flow offsets the flow recording and for further processing the offset must be subtracted. As a result the technique will be more vulnerable to artifacts, non-linearity and drift. Without bias flow (in-line placement) these errors will be less obvious since breathing will be symmetrical around zero flow.

### Studies using flow through technique

Lahiri et al used the flow through technique to investigate newborn infants responses to O2 and CO2 at high altitudes [10]. Ruttimann et al added a second pneumotach on the inspiratory tubing and tested this in animals [11]. Thomson et al also used flow-through technique with two pneumotachographs to determine work of breathing in intubated infants with spontaneous breathing [12]. They also used the system with a face mask in healthy neonates (mean weight 1230 g). The use of double pneumotachographs has been further studied and has the advantage of allowing variations in bias-flow [13]. Measuring the bias-flow with a second flow meter increases the complexity of the flow-through technique. It is used in modern ventilators (bias-flow adjusted with the inspiratory valve) on adults and children but in infants with lower airway flows it is increasingly problematic and the use of in-line placement of an external flow meter is common.

The flow-through technique has also been used with CPAP. In a cross-over comparison by Huckstadt et al used pneumotachgraphs for measurements with two CPAP systems (Infant Flow variable flow nasal CPAP and Babylog 8000 ventilator) [14]. They reported pressure stability for the two CPAP systems and measurements were performed with a Baby pneumotachograph (Jaeger, Germany) with a low flow resistance (0.034 cm H2O /(L/min)).

Andréasson et al compared lung function variables with and without CPAP after extubation [15]. The CPAP was generated with a resistor, measured with a Fleisch no. 2, and applied using a face chamber. In a recent study flow-through measurements were used in combination with wash-out to determine FRC during CPAP [16].

The technique has also been used in positive pressure ventilation [12, 17]. The effect of pressure swings in the circuit makes flow measurements challenging since they are affected by gas compression as well as the compliance and resistance of the tubing used. The problem with gas compression and tube compliance should be smaller in situations with smaller pressure swings, for example during spontaneous breathing and pressure stable NCPAP systems.

### Limitations and problems

The flow meters in our study are used for measuring flows in a range between -6 and 16 L/min. This may be outside what the flow meters were designed and optimized for but the range is relevant for use in the neonatal setting.

The sinusoidal breath profile is artificial and very far from normal breathing patterns. It was preferred for two reasons; it is symmetrical and allows graphical comparisons of recorded flows and it is easy for others to reproduce.

The quality of the flow recording is presented graphically. This is a crude way to present the recorded flow and signal quality. Further processing (for example integration to calculate tidal volume) would not make the comparison easier and would have needed complex decisions on how to process the signals including choice of filters and corrections of non-linearity. The level of processing varies between the devices; some of the recorded signals have already been processed (e.g. FLORIAN) while others are unprocessed (e.g. Fleisch pneumotachographs). We therefore chose to present the signals without any further processing. It may be possible to improve signal quality for some of the devices.

In clinical use there are several aspects of performance that are important but were not tested in this study. These include drift, need for calibration and recalibration, effects from gas composition and temperature (including humidity and oxygen fraction).

### Clinical implications and future directions

The effects of imposed WOB have been insufficiently studied. If low imposed WOB during NCPAP is important, results from previous trials using high imposed WOB NCPAP may not be applicable to devices with low imposed WOB. This will also be true for clinical studies of lung function variables that have been recorded with high imposed WOB equipment.

A flow meter that has little or no effect on imposed work of breathing and sufficiently high accuracy would be a useful tool in clinical and experimental studies. The two flow meters dedicated for neonatal use have a resistance that will add to imposed WOB. This means that these are not optimal for some experiments or investigations. Reducing resistance will be particularly important when investigating variables that may depend on imposed work of breathing and flow resistance.

Two of the tested devices (SFM3200 and the Vitalograph Fleisch pneumotachograph) show low resistance and a graphic quality of the flow signal that is sufficiently high to be interesting for further evaluation. The overall aim of developing these techniques would be to be able to measure flows without adding resistance or dead space during NCPAP treatment of neonates.

## Conclusions

The tested flow meters showed increased imposed WOB with increasing resistance in both the in-line and flow-through position. In simulations with bias flow the resistance also generated CPAP pressure. Our results can be used to evaluate imposed WOB for existing and new systems. It should also stimulate further research for development of equipment for dynamic flow measurements in neonates. The results indicate that the flow-through technique may be a solution for obtaining a sufficiently high signal quality without the problems of rebreathing and increased work of breathing.

## Supporting Information

S1 Table Mean pressure and imposed WOB (mean, SD) for simulations with bias flow. Data from twenty consecutive breaths, using 16 ml or 32 ml tidal volume. A bias flow of 0 L/min is equivalent to in-line placement of flow meter. Letters a-H indicate p>0.05 in one or more comparisons.
(PDF)

S2 Table Mean pressure and imposed WOB (mean, SD) for simulation with Infant Flow at three levels of CPAP. Data from twenty consecutive breaths, using 32 ml tidal volume. The ‘No Flow Meter’ row represents baseline measurement of the Infant Flow system tested without a flow meter attached to the exhaust limb. Letters a-w indicate p>0.05 in one or more comparisons.
(PDF)
