# Supplementary material for: Imposed Work of Breathing for Flow Meters with In-Line versus Flow-Through Technique during Simulated Neonatal Breathing
Source: PLoS One. 2015 Jul 20;10(7):e0133432. doi: 10.1371/journal.pone.0133432 (PMC4507850; doi:10.1371/journal.pone.0133432)
Supplement: S1 Table — Data from twenty consecutive breaths, using 16 ml or 32 ml tidal volume. A bias flow of 0 L/min is equivalent to in-line placement of flow meter. Letters a-H indicate p>0.05 in one or more comparisons. (PDF) [file pone.0133432.s001.pdf]

			CPAP (cm H2O)		P decrease (cm H2O)		P increase (cm H2O)		Total iWOB (mJ/breath)		Insp iWOB (mJ/breath)		Exp iWOB (mJ/breath)	
			Mean	SD	Mean	SD	Mean	SD	Mean	SD	Mean	SD	Mean	SD
16 ml	0 l/min	SpiroQuant A	-0,01	0,00	0,06 [e]	0,03	0,04 [l]	0,01	0,01	0,00	0,01 [v]	0,02	-0,01	0,02
		SFM3200 prototype	0,00	0,00	0,06 [e]	0,02	0,05	0,01	0,04 [s]	0,00	0,01 [v]	0,03	0,03 [B]	0,03
		Vitalograph Fleisch	-0,01 [a]	0,00	0,05 [e]	0,02	0,04 [l]	0,01	0,05 [s]	0,00	0,02 [v]	0,01	0,03 [B]	0,02
		Fleisch 0	0,00	0,00	0,23 [f]	0,02	0,23 [m]	0,01	0,51	0,00	0,25 [w]	0,01	0,26	0,01
		FLORIAN	-0,01 [a]	0,00	0,23 [f]	0,01	0,23 [m]	0,01	0,46	0,00	0,23 [w]	0,01	0,23 [C]	0,01
		EXHALYZER S	-0,01 [a]	0,00	0,26	0,01	0,23 [m]	0,01	0,47	0,01	0,24 [w]	0,01	0,23 [C]	0,01
	5 l/min	SpiroQuant A	-0,01	0,00	0,06 [g]	0,03	0,04 [n]	0,01	0,01	0,00	0,01	0,02	0,01 [D]	0,02
		SFM3200 prototype	0,02 [b]	0,00	0,08	0,02	0,05 [n]	0,01	0,05 [t]	0,00	0,03 [x]	0,02	0,02 [D]	0,02
		Vitalograph Fleisch	0,02 [b]	0,00	0,06 [g]	0,01	0,05 [n]	0,01	0,06 [t]	0,00	0,03 [x]	0,01	0,03 [D]	0,01
		Fleisch 0	0,35	0,00	0,24	0,01	0,26	0,01	0,56	0,01	0,27	0,01	0,29	0,01
		FLORIAN	0,50	0,00	0,40	0,02	0,48	0,01	1,02	0,01	0,44	0,01	0,58	0,01
		EXHALYZER S	0,60	0,00	0,49	0,01	0,63	0,01	1,33	0,01	0,53	0,01	0,79	0,01
	10 l/min	SpiroQuant A	-0,01	0,00	0,06 [h]	0,02	0,05 [o]	0,01	0,03	0,00	0,01	0,03	0,02 [E]	0,03
		SFM3200 prototype	0,04	0,00	0,07 [h]	0,02	0,05 [o]	0,01	0,06	0,00	0,03 [y]	0,02	0,03 [E]	0,02
		Vitalograph Fleisch	0,07	0,00	0,05 [h]	0,02	0,04 [o]	0,01	0,06	0,01	0,03 [y]	0,01	0,03 [E]	0,01
		Fleisch 0	0,82	0,00	0,27	0,02	0,28	0,01	0,63	0,01	0,31	0,01	0,32	0,01
		FLORIAN	1,45	0,00	0,68	0,02	0,75	0,01	1,66	0,01	0,76	0,01	0,90	0,01
		EXHALYZER S	1,82	0,00	0,92	0,04	1,04	0,02	2,32	0,01	1,05	0,02	1,27	0,03
32 ml	0 l/min	SpiroQuant A	-0,01	0,00	0,06 [i]	0,02	0,05	0,01	0,04	0,01	0,01	0,02	0,03	0,02
		SFM3200 prototype	-0,02 [c]	0,00	0,08 [i]	0,01	0,07 [p]	0,01	0,16	0,01	0,08 [z]	0,04	0,08 [F]	0,04
		Vitalograph Fleisch	-0,03	0,00	0,07 [i]	0,02	0,06 [p]	0,01	0,19	0,01	0,09 [z]	0,03	0,09 [F]	0,03
		Fleisch 0	-0,02 [c]	0,00	0,46	0,02	0,45	0,01	2,14	0,01	1,07	0,02	1,06	0,02
		FLORIAN	0,01	0,00	0,61	0,02	0,61	0,01	2,65	0,01	1,31	0,02	1,34	0,02
		EXHALYZER S	-0,02	0,00	0,79	0,02	0,76	0,01	3,21	0,02	1,65	0,03	1,56	0,03
	5 l/min	SpiroQuant A	0,01	0,00	0,07 [j]	0,02	0,05 [q]	0,01	0,06	0,01	0,01	0,03	0,05	0,03
		SFM3200 prototype	0,00 [d]	0,00	0,08 [j]	0,02	0,07 [q]	0,01	0,18	0,01	0,07	0,04	0,11 [G]	0,04
		Vitalograph Fleisch	0,00 [d]	0,00	0,07 [j]	0,01	0,06 [q]	0,01	0,20	0,01	0,10	0,02	0,10 [G]	0,02
		Fleisch 0	0,38	0,00	0,47	0,02	0,50	0,01	2,29	0,01	1,09	0,02	1,20	0,03
		FLORIAN	0,61	0,00	0,68	0,01	1,02	0,01	4,09	0,01	1,39	0,02	2,71	0,02
		EXHALYZER S	0,81	0,00	0,86	0,02	1,40	0,01	5,41	0,01	1,63	0,03	3,78	0,03
	10 l/min	SpiroQuant A	0,03	0,00	0,07	0,02	0,05	0,01	0,10	0,01	0,04	0,05	0,06	0,05
		SFM3200 prototype	0,05	0,00	0,09 [k]	0,02	0,08 [r]	0,01	0,24 [u]	0,01	0,13 [A]	0,05	0,11 [H]	0,05
		Vitalograph Fleisch	0,07	0,00	0,08 [k]	0,01	0,07 [r]	0,01	0,23 [u]	0,01	0,13 [A]	0,02	0,11 [H]	0,02
		Fleisch 0	0,78	0,00	0,51	0,01	0,55	0,01	2,52	0,01	1,20	0,02	1,32	0,02
		FLORIAN	1,55	0,00	1,23	0,01	1,54	0,01	6,71	0,01	2,80	0,02	3,91	0,02
		EXHALYZER S	2,11	0,00	1,73	0,02	2,24	0,02	9,63	0,02	3,94	0,03	5,69	0,03

S1 Table. Mean pressure and imposed WOB (mean, SD) for simulations with bias flow. Data from twenty consecutive breaths, using 16 ml or 32 ml tidal volume. A bias flow of 0 l/min is equivalent to in-line placement of flow meter. Letters a-H indicate p>0.05 in one or more comparisons.
