# Supplementary material for: Imposed Work of Breathing for Flow Meters with In-Line versus Flow-Through Technique during Simulated Neonatal Breathing
Source: PLoS One. 2015 Jul 20;10(7):e0133432. doi: 10.1371/journal.pone.0133432 (PMC4507850; doi:10.1371/journal.pone.0133432)
Supplement: S2 Table — Data from twenty consecutive breaths, using 32 ml tidal volume. The ‘No Flow Meter’ row represents baseline measurement of the Infant Flow system tested without a flow meter attached to the exhaust limb. Letters a-w indicate p>0.05 in one or more comparisons. (PDF) [file pone.0133432.s002.pdf]

		CPAP (cm H2O)		P decrease (cm H2O)		P increase (cm H2O)		Total iWOB (cm H2O)		Insp iWOB (cm H2O)		Exp iWOB (cm H2O)	
CPAP	System	Mean	SD	Mean	SD	Mean	SD	Mean	SD	Mean	SD	Mean	SD
3 cm H2O	No Flow Meter	2,93	0,01	0,58 [c]	0,03	0,47 [f]	0,02	2,17 [j]	0,03	1,34 [n]	0,05	0,83 [s]	0,04
	SpiroQuant A	2,95	0,01	0,60 [c]	0,04	0,47 [f]	0,02	2,16 [j]	0,03	1,34 [n]	0,04	0,82 [s]	0,04
	SFM3200 prototype	2,94	0,01	0,62 [c]	0,04	0,50 [g]	0,01	2,41	0,03	1,44 [o]	0,04	0,97 [t]	0,05
	Vitalograph Fleisch	2,97	0,00	0,62 [c]	0,02	0,50 [g]	0,02	2,38	0,02	1,43 [o]	0,03	0,94 [t]	0,04
	Fleisch 0	2,95	0,01	0,94	0,04	0,79	0,02	3,78	0,03	2,14	0,06	1,64	0,06
	FLORIAN	3,05	0,01	1,17	0,04	1,19	0,02	5,39	0,03	2,52	0,02	2,87	0,04
	EXHALYZER S	3,13	0,00	1,29	0,04	1,55	0,02	6,50	0,02	2,66	0,05	3,84	0,06
5 cm H2O	No Flow Meter	5,03 [a]	0,01	0,65 [d]	0,03	0,69 [h]	0,03	2,98 [k]	0,06	1,49 [p]	0,05	1,48 [u]	0,08
	SpiroQuant A	5,02 [a]	0,01	0,65 [d]	0,03	0,71 [h]	0,04	3,00 [k]	0,06	1,53 [p]	0,05	1,46 [u]	0,06
	SFM3200 prototype	5,03 [a]	0,01	0,70 [d]	0,04	0,72 [h]	0,06	3,16 [l]	0,06	1,59 [p]	0,05	1,57 [v]	0,08
	Vitalograph Fleisch	5,08	0,01	0,69 [d]	0,03	0,74 [h]	0,06	3,17 [l]	0,06	1,57 [p]	0,05	1,59 [v]	0,08
	Fleisch 0	5,02 [a]	0,01	0,97	0,04	0,90	0,05	4,25	0,10	2,19	0,05	2,07	0,09
	FLORIAN	5,14	0,01	1,36	0,04	1,48	0,05	6,70	0,08	2,80	0,05	3,90	0,09
	EXHALYZER S	5,17	0,01	1,52	0,03	1,78	0,05	7,87	0,07	3,27	0,05	4,60	0,08
8 cm H2O	No Flow Meter	8,14 [b]	0,01	0,90 [e]	0,05	1,15 [i]	0,06	4,80	0,07	1,85 [q]	0,06	2,95 [w]	0,08
	SpiroQuant A	8,19	0,01	0,91 [e]	0,03	1,16 [i]	0,05	4,89 [m]	0,08	1,89 [q]	0,06	2,99 [w]	0,09
	SFM3200 prototype	8,15	0,01	0,92 [e]	0,04	1,17 [i]	0,05	5,03 [m]	0,07	1,96 [q]	0,06	3,07 [w]	0,07
	Vitalograph Fleisch	8,14 [b]	0,01	0,91 [e]	0,04	1,16 [i]	0,04	4,96 [m]	0,08	1,92 [q]	0,05	3,04 [w]	0,09
	Fleisch 0	8,21	0,01	1,14	0,05	1,36	0,05	5,90	0,07	2,29	0,06	3,61	0,09
	FLORIAN	8,25	0,01	1,56	0,06	1,96	0,06	8,20	0,10	3,22	0,09	4,98	0,16
	EXHALYZER S	8,33	0,01	1,91	0,06	2,43	0,05	10,40	0,07	3,97	0,07	6,43	0,10

S2 Table. Mean pressure and imposed WOB (mean, SD) for simulation with Infant Flow at three levels of CPAP. Data from twenty consecutive breaths, using 32 ml tidal volume. The ‘No Flow Meter’ row represents baseline measurement of the Infant Flow system tested without a flow meter attached to the exhaust limb. Letters a-w indicate p>0.05 in one or more comparisons.
